# The importance and application of the ancestral recombination graph

**DOI:** 10.3389/fgene.2013.00206

**Published:** 2013-10-14

**Authors:** Miguel Arenas

**Affiliations:** Centre for Molecular Biology “Severo Ochoa,” Consejo Superior de Investigaciones Científicas, Universidad Autónoma de MadridMadrid, Spain

**Keywords:** ancestral recombination graph, recombination network, recombination breakpoints, ancestral material, recombinant fragment, phylogenetic bias

One of the most important evolutionary forces is recombination, it increases genetic diversity and promotes adaptation through exchange of genetic material and where existent mutations are shuffled. Knowledge about recombination is, for example, fundamental to understand genome structure (Reich et al., 2001), phenotypic diversity (Zhang et al., 2002), and diverse genetic diseases (Daly et al., 2001). Indeed, recombination should be considered to properly study molecular evolution and perform phylogenetic inferences (e.g., Schierup and Hein, 2000; Anisimova et al., 2003; Arenas and Posada, 2010c). The recombination evolutionary history is commonly represented by the ancestral recombination graph (ARG) (Griffiths and Marjoram, 1997), an illustrative example is shown in Figure 1. Counterintuitively, ARGs have not been widely used, perhaps as a consequence of the difficulties to infer explicit ARGs and the complexity of the ARG representation. The aim of this general commentary is to describe the importance and application of the ARG.

**Figure 1:**
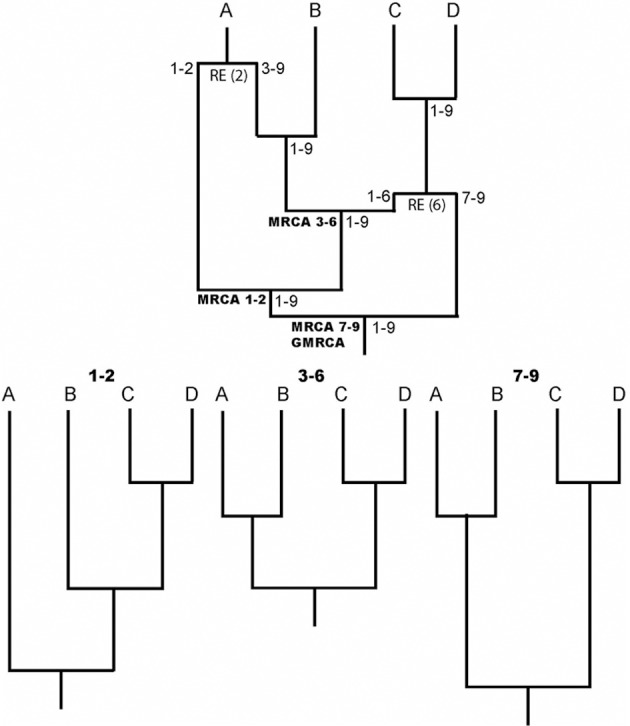
**Illustrative example of an ARG.** RE indicates recombination events. Numbers in nodes indicate intervals of ancestral material. Note that each recombinant fragment (1–2, 3–6, and 7–9) has its own most recent common ancestor (MRCA), all of which finally coalesce into a grand MRCA (GMRCA). At the bottom, the trees corresponding to each recombinant fragment are shown.

## Working with the ARG

A key aspect of ARG software is the ARG format. Notice that the description of an ARG is not straightforward because it is not a simple phylogenetic network and therefore, it cannot be represented by a classic network format [e.g., Extended Newick; see Arenas et al. (2010)]. Any ARG format must include not only the evolutionary history, but also the regions with ancestral material (material that reaches the sample) for each node (see Figure 1). Only a few ARG formats have been developed so far (Buendia and Narasimhan, 2006; Vos et al., 2012) and there is a need for a standard format that would allow an easy communication among different ARG software. In this regard, recently McGill et al. (2013) presented the *ArgML* format that is based on XML syntax and can include detailed information about the ARG (e.g., recombination breakpoints and ancestral material). These features suggest *ArgML* as a successful candidate to the standard ARG format.

Several methods and computer tools exist to simulate and infer ARGs.

From the simulation perspective, to my knowledge, only two programs can output a simulated ARG, namely, *Serial NetEvolve* (Buendia and Narasimhan, 2006) and *NetRecodon* (Arenas and Posada, 2010a). Most of simulation tools only simulate a tree for each recombinant fragment, but these trees can be then combined to generate the ARG by using tools like *CombineTrees* (see Woolley et al., 2008).

On the other hand, the inference of ARGs is complex and computationally extensive (see Rasmussen and Siepel, 2013 and references therein). For this reason, a common procedure to infer an ARG consists of the detection of recombination breakpoints (see Martin et al., 2011) followed by a phylogenetic tree reconstruction for each recombinant fragment and finally, a combination of all the reconstructed trees. Actually, the *IRiS* tool (Javed et al., 2011) has automated this whole procedure described above. However, in recent years, new methods to directly infer ARGs are emerging. For example, *ACG* (O'Fallon, 2013) that is based on a Bayesian Markov chain Monte Carlo (MCMC) procedure to compute the full likelihood of the ARG, or *ARGweaver* (Rasmussen and Siepel, 2013) that is based on hidden Markov models (HMM) and can infer ARGs from genome-wide data.

## Applying the ARG

ARGs can be applied for a variety of purposes, some examples are described below.

1. Detailed visualization and understanding of relationships among lineages, timing of recombination events and genetic exchange (e.g., Utro et al., 2012).
2. The ARG can be used in a variety of population genetics inferences related with demographics, population divergence times, migration and selection. Note that these population genetics scenarios may affect the length and shape of the ARG (Hudson and Kaplan, 1988; Griffiths and Marjoram, 1997; Rasmussen and Siepel, 2013). For example, it is known that variable population sizes can alter the branch lengths, in particular coalescences are more likely to occur when the population is small. Therefore, the ARG can be used to find correlations between recombination rate and population size over time (e.g., Birkner et al., 2013).
3. Recombination can bias phylogenetic tree reconstruction (e.g., Schierup and Hein, 2000; Posada and Crandall, 2002) and derived inferences (Anisimova et al., 2003; Arenas and Posada, 2010a,b,c). In order to avoid such a bias, trees embedded in the ARG can be used to correctly perform phylogenetic estimations (e.g., ancestral sequence reconstruction or molecular adaptation) accounting for recombination (e.g., Perez-Losada et al., 2009, 2011).
4. Molecular evolution can be simulated over an ARG (e.g., Buendia and Narasimhan, 2006) and the simulated genetic data can be applied for hypothesis testing, to evaluate analytical tools (see the reviews Arenas, 2012, 2013) or to estimate evolutionary parameters by using approaches like approximate Bayesian computation (ABC) (Wilson et al., 2009; Lopes et al., in press).

## Conclusions

The ARG is indispensable to study evolutionary scenarios where recombination has occurred. With the development of next-generation sequencing (NGS) technologies there is a growing number of genomes at our disposal, many of which could have evolved under recombination (e.g., Utro et al., 2012; Rasmussen and Siepel, 2013). As a consequence, the importance and application of ARGs is expected to increase over the next years.

In order to make progress in the use of ARGs, a key aspect is the design of a standard format to represent the ARG. In this regard, McGill et al. (2013) presented a format based on XML syntax that can easily be used to store and communicate ARGs.
